# Corrigendum to: A coevolved EDS1-SAG101-NRG1 module mediates cell death signaling by TIR-domain immune receptors

**DOI:** 10.1093/plcell/koab085

**Published:** 2021-04-01

**Authors:** Dmitry Lapin, Viera Kovacova, Xinhua Sun, Joram A Dongus, Deepak Bhandari, Patrick von Born, Jaqueline Bautor, Nina Guarneri, Jakub Rzemieniewski, Johannes Stuttmann, Andreas Beyer, Jane E Parker

Plant Cell (2019) 31: 2430–2455 doi: 10.1105/tpc.19.00118

The primer combination DB115+DB116 used to generate a mutant of Arabidopsis *EDS1* (*AtEDS1*) was labeled "H476F" instead of "H476Y" in our stocks. This error was overlooked during sequence validation of constructs described in the manuscript. The DB115 and DB116 sequences provided in Supplemental Table 3 correspond to the H476Y and not H476F exchange (see the translation below).

DB115 (and reverse DB116) primers in Supplemental Table 3:

caaactaccatcga**tat**ttaaagaacgaag

N Y H R **Y** L K N E

The conclusions in our study are not affected since we did not test effects of other substitutions of H476 in *AtEDS1*. In all instances, “H476F” should, however, be read as “H476Y”. We apologize for the error. All authors agreed to this correction.

We further note that in a follow-up study by Sun and colleagues (Sun et al. “Pathogen effector recognition-dependent association of NRG1 with EDS1 and SAG101 in TNL receptor immunity”. bioRxiv, 2020.2012.2021.423810), primers DB115 and DB116 for the *AtEDS1* H476Y mutation are presented correctly and corresponding constructs have been sequence-validated.

Editors’ note: This correction was reviewed by members of The Plant Cell editorial board. The authors are responsible for providing a complete listing and accurate explanations for all known errors or instances of inappropriate data handling or image manipulation associated with the original publication.

